# Possible role of arbuscular mycorrhizal fungi and associated bacteria in the recruitment of endophytic bacterial communities by plant roots

**DOI:** 10.1007/s00572-021-01040-7

**Published:** 2021-07-20

**Authors:** Gergely Ujvári, Alessandra Turrini, Luciano Avio, Monica Agnolucci

**Affiliations:** Department of Agriculture, Food and Environment, University of Pisa, 56124 Pisa, Italy

**Keywords:** AMF-associated bacteria, Composition of root bacterial communities, Plant growth–promoting bacteria, Mycorrhizal inoculum, Biological soil fertility

## Abstract

Arbuscular mycorrhizal fungi (AMF) represent an important group of root symbionts, given the key role they play in the enhancement of plant nutrition, health, and product quality. The services provided by AMF often are facilitated by large and diverse beneficial bacterial communities, closely associated with spores, sporocarps, and extraradical mycelium, showing different functional activities, such as N_2_ fixation, nutrient mobilization, and plant hormone, antibiotic, and siderophore production and also mycorrhizal establishment promotion, leading to the enhancement of host plant performance. The potential functional complementarity of AMF and associated microbiota poses a key question as to whether members of AMF-associated bacterial communities can colonize the root system after establishment of mycorrhizas, thereby becoming endophytic. Root endophytic bacterial communities are currently studied for the benefits provided to host plants in the form of growth promotion, stress reduction, inhibition of plant pathogens, and plant hormone release. Their quantitative and qualitative composition is influenced by many factors, such as geographical location, soil type, host genotype, and cultivation practices. Recent data suggest that an additional factor affecting bacterial endophyte recruitment could be AMF and their associated bacteria, even though the mechanisms allowing members of AMF-associated bacterial communities to actually establish in the root system, becoming endophytic, remain to be determined. Given the diverse plant growth–promoting properties shown by AMF-associated bacteria, further studies are needed to understand whether AMF may represent suitable tools to introduce beneficial root endophytes in sustainable and organic agriculture where the functioning of such multipartite association may be crucial for crop production.

## Introduction

In recent years, the rising demand for the production of environmentally safe and high-quality food has caused a major shift in agricultural management, which is growing increasingly sustainable by making use of practices able to maintain and enhance soil fertility and health (FAO 2011). In this context, soil microorganisms play a key role in the modulation of soil biochemical, biological, and nutritional processes (Azcón-Aguilar and Barea 2015). Such microbiota thrives in a privileged niche at the soil–root interface (rhizosphere) that is a rich source of nourishment, represented by sugars, amino acids, and organic acids in the form of root exudates (Philippot et al. 2013). The complex microbial communities establishing in the rhizosphere have profound effects on plant growth, nutrition, and health (Compant et al. 2010; Hayat et al. 2010). Members of the rhizospheric microbiota may establish an intimate relationship with their host plants, colonizing roots and also aboveground plant compartments, becoming endophytes, i.e., microorganisms which can be isolated from, or detected within, surface-sterilized plant organs and do not cause visible harm to the host organism (Hardoim et al. 2008). Bacterial endophytes of plant roots may reach a density of 10^4^–10^8^ bacterial cells per gram of root tissue and may have important roles in plant growth promotion, fitness, and protection against pathogens. In exchange, the plant endosphere provides the endophytic microbiota with a more uniform, protected, and nutrient-rich environment than the nearby soil (Hardoim et al. 2015; Liu et al. 2017b). The use of green fluorescent protein labeling, image analysis, and fluorescence in situ hybridization–confocal laser scanning microscopy has allowed the localization of bacterial endophytes in intercellular spaces, at the base of lateral roots and root tips, in xylem vessels, and inside root hairs (Compant et al. 2010; Gaiero et al. 2013; Liu et al. 2017a).

Arbuscular mycorrhizal fungi (AMF) represent an important group of root endophytes in view of their key role in the enhancement of plant nutrition, health, and product quality. AMF (phylum Glomeromycota) are beneficial soil fungi, establishing mutualistic symbioses with about 80% of land plants including the major food crops, such as cereals, pulses, vegetables, and fruit trees and industrial crops like cotton, sunflower, and oil palm (Smith and Read 2008). They are obligate biotrophic organisms which obtain carbon from the host plant, providing in exchange soil mineral nutrients (such as P, N, S, K, Ca, Cu, and Zn), absorbed and translocated by the extraradical mycelium (ERM) extending from colonized roots into the soil. Therefore, the ERM represents an efficient auxiliary absorbing system because of its high surface-to-volume ratio, hyphal P absorption beyond the P depletion zone around roots, and the occurrence of many nutrient transporters in its hyphae (Smith and Read 2008; Pepe et al. 2017; Kameoka et al. 2019). Moreover, AMF improve plant performance and health by increasing plant tolerance to biotic and abiotic stresses (Sikes et al. 2009; Bitterlich et al. 2018) and induce changes in plant secondary metabolism leading to enhanced biosynthesis of health-promoting phytochemicals (Avio et al. 2018; Agnolucci et al. 2020). Overall, AMF provide multifunctional ecosystem services and are utilized as biofertilizers, biostimulants, and bioenhancers in agriculture (Gianinazzi et al. 2010; Rouphael et al. 2015).

Arbuscular mycorrhizal fungi live closely associated with large and diverse bacterial communities which may colonize spores, sporocarps, and extraradical hyphae, originating a complex and metabolically active environment called the mycorrhizosphere (Rambelli 1973). Such microbiota show different plant growth–promoting properties (plant hormone, antibiotic, and siderophore production; N_2_ fixation; P solubilization) and mycorrhiza helper activities (spore germination and mycelial growth promotion, mycorrhizal establishment facilitation) also have been observed, leading to enhancement of host plant performance (Bharadwaj et al. 2008a; Battini et al. 2016; 2017; Sharma et al. 2020). The potential functional complementarity and synergistic activity of AMF and their associated microbiota necessitate studies aimed at understanding the complex network of interactions between them and their host plants (Turrini et al. 2018). This is all the more important when implementing AMF inocula in sustainable and organic agriculture where the functioning of such multipartite associations may be crucial for crop production. Notwithstanding, scanty information is available on the relationship between AMF-associated bacteria and the bacterial microbiota colonizing roots after AMF inoculation. Here, we (i) provide an overview of recent developments regarding the recruitment of root endophytic bacteria, (ii) present data on the diversity and functionality of AMF-associated bacterial communities, and (iii) discuss the possible role of AMF in shaping the structure and composition of endophytic bacterial communities recruited by plant roots.

## Endophytic bacteria recruited by plant roots

### Characteristics and importance of root endophytic bacteria

Bacterial endophytes are able to recognize plant root exudates, adhere to the root surface, form a biofilm, and then enter roots, colonizing their inner tissues. According to current knowledge, passive penetration can take place at wounds, root cracks, secondary root emergence points, and root tips, while active colonization can involve cell wall–degrading hydrolytic enzymes (Compant et al. 2010). The presence of flagella, pili, lipopolysaccharides, exopolysaccharides, some special membrane proteins, quorum-sensing signals, chemotactic abilities, protein secretion systems, and twitching motility also may have importance in the invasion processes of certain endophytic bacteria, as shown by comparative genome analyses (Sessitsch et al. 2012; Hardoim et al. 2015; Pinski et al. 2019). Some of the required endophytic/symbiotic genes may be coded on plasmids or “symbiosis islands,” suggesting the possibility of horizontal transfer of these functional genes among the members of soil bacterial communities (Finan 2002). Nevertheless, vertical ways of endophyte transmission (seed-borne endophytes) also have been confirmed (Truyens et al. 2015) in which cases the transmitted bacteria were able to colonize the rhizospheres of new plantlets (Kaga et al. 2009; Hameed et al. 2015).

Bacteria colonizing the root endosphere profit from enhanced nutrient availability and environmental homeostasis provided by the plant, while the host plant may receive benefits from the endophytes in the form of direct and indirect growth promotion, stress reduction, or inhibition of plant pathogens (Gaiero et al. 2013; Hardoim et al. 2008; 2015; Compant et al. 2010; Pinski et al. 2019). As to nutrient mobilization, many endophytic bacteria were reported to possess N_2_-fixing, nitrifying, denitrifying, and P-solubilizing abilities or to be able to produce siderophores (Sessitsch et al. 2012; Hameed et al. 2015). Bacterial root endophytes influence plant hormone levels directly or indirectly, affecting growth, stress, and immune responses of host plants. Many endophytes may be able to release indoleacetic acids (IAA), gibberellins, cytokinins, ethylene, abscisic acid, jasmonates, and volatile compounds, while some of them also act as a sink for 1-aminocyclopropane-1-carboxylate (ACC), a precursor of ethylene production, because of a cytoplasmic ACC deaminase enzyme. The features mentioned suggest that bacterial endophytes may have a fundamental role in “fine-tuning” the hormonal balance of the host plant (Forchetti et al. 2007; Glick 2014; Hardoim et al. 2015). Furthermore, a key feature of endophytic strains is detoxification of reactive oxygen species, reactive nitrogen species, glutathione synthases, and glutathione-S-transferases, ameliorating different effectors of plant stress responses (Sessitsch et al. 2012). In the unique ecological niche where they thrive, some root endophytic bacteria may produce bioactive secondary metabolites, such as antibiotic and antiviral compounds (Strobel et al. 2004; Ryan et al. 2008; Ek-Ramos et al. 2019), while others have shown potential for enhancement of phytoremediation procedures because of their ability to decompose contaminants (Ryan et al. 2008; Mitter et al. 2019). Some authors have reported that the root microbiota may contribute to improved food quality through biofortification or production of health-promoting metabolites (Rehman et al. 2018; Ku et al. 2019) and potentially affect the quality of processed food products. For example, Minervini et al. (2015) demonstrated that endophytic lactic acid bacteria occurred not only in roots and various organs of durum wheat plants (*Triticum turgidum* ssp. *durum*) but also in the flour, possibly inducing changes in microbial community structure and properties of sourdough and derived products. Accordingly, the isolation and selection of functional root endophytic bacterial strains may be of particular interest for agriculture, industrial biotechnology, and medicine.

### Recruitment of bacterial root endophytes by host plants

The quantitative and qualitative composition of root endophytic bacterial communities is influenced by many factors, mainly by geographical location, soil source, host genotype, and cultivation practice (Edwards et al. 2015). In Table 1, data from recent metagenomic studies of endophytic bacteria are reported. During notable work of defining the core root microbiome of the non-mycorrhizal species *Arabidopsis thaliana* L., based on 16S rDNA high-throughput sequencing data, two research groups revealed that the composition of root endophytic bacterial communities may be influenced by host genotype (Bulgarelli et al. 2012; Lundberg et al. 2012). Other studies demonstrated that the host plant may modulate the occurrence of root microbiota recruited from the nearby soil environment. Manter et al. (2010), utilizing a pyrosequencing approach, investigated the root endophytic communities of 20 potato cultivars and clones, revealing significant differences among the taxonomic profiles within those different plant genotypes. In that study, the identified bacterial operational taxonomic units (OTUs) affiliated with 238 genera and 15 phyla, demonstrating the remarkable diversity and variability of root endophytes. Plant genotype was found to shape the community composition of bacteria associated with the roots of 10 different rice cultivars (Hardoim et al. 2011), while differences in the bacterial root microbiota in the non-mycotrophic Brassicaceae family were found to be largely quantitative (Schlaeppi et al. 2014). Using the Illumina MiSeq platform, Marasco et al. (2018) found that grapevine (*Vitis vinifera* L.) rootstock genotypes influenced the taxonomic composition of their endophytic bacterial communities, although plant growth–promoting traits were not significantly different among the cultivars, showing a homeostasis of the plant/bacterial endophyte relationship. Culture-independent techniques, PCR-denaturing gradient gel electrophoresis (DGGE) and Illumina MiSeq sequencing of the 16S rDNA of root endophytic bacterial communities confirmed the selectivity of genotypes in durum wheat, as different cultivars hosted significantly different bacterial communities in their root tissues (Agnolucci et al. 2019b). However, Singer et al. (2019) found conserved community structures across different genotypes of *Panicum virgatum* and *Panicum hallii*.

**Table 1:** Relative abundance of the most represented bacterial phyla in the root endophytic communities of host plants from different ecosystems and geographic locations, as assessed by metagenomic approaches. In each study, only bacterial phyla with a relative abundance ≥ 2% were considered

Plant order	Host plant species	Geographic location	Site/soil characteristics	Methodology	Target region (16S rDNA)	Endophytic bacterial community composition^a^		Reference
Asparagales	*Agave tequilana, A. salmiana *and* A. deserti*	Mexico and USA	clay/clay loam/sandy loam agricultural and natural sites	Illumina MiSeq	V4	Proteobacteria	52%	Coleman-Derr et al. (2016)
						Actinobacteria	31%	
						Bacteroidetes	10%	
						Firmicutes	4%	
	*Aloe vera*	Malaysia	horticultural nursery	Illumina MiSeq	V3-V4	Proteobacteria	35%	Akinsanya et al. (2015)
						Firmicutes	17%	
						Actinobacteria	11%	
						Bacteroidetes	10%	
	*Dendrobium officinale*	China	field soil	Illumina MiSeq	V4	Proteobacteria	77%	Pei et al. (2017)
						Actinobacteria	19%	
						Bacteroidetes	2%	
						Firmicutes	2%	
Brassicales	*Arabidopsis thaliana *^**b**^	USA	pesticide-free agricultural soils	Roche 454	V7-V8	Actinobacteria	48%	Lundberg et al. (2012)
						Proteobacteria	22%	
						Bacteroidetes	13%	
						Cyanobacteria	8%	
						Firmicutes	7%	
	*Arabidopsis thaliana *^**b**^	USA	disturbed sites	Roche 454	V5-V7	Proteobacteria	45%	Bodenhausen et al. (2013)
						Actinobacteria	31%	
						Bacteroidetes	22%	
	*Arabidopsis thaliana *^**b**^	Germany	chemical-free research field soils	Roche 454	V5-V7	Proteobacteria	49%	Bulgarelli et al. (2012)
						Actinobacteria	26%	
						Bacteroidetes	9%	
						Planctomycetes	4%	
						Saccharibacteria	4%	
						Acidobacteria	2%	
	*Arabidopsis thaliana *^**b**^	Germany	natural and research field sites	Roche 454	V5-V7	Proteobacteria	45%	Schlaeppi et al. (2014)
						Actinobacteria	23%	
						Bacteroidetes	20%	
						Dormibacteraeota	4%	
						Chloroflexi	2%	
	*Cardamine hirsuta*					Proteobacteria	48%	
						Actinobacteria	27%	
						Bacteroidetes	10%	
						Firmicutes	5%	
						Chloroflexi	2%	
Caryophyllales	*Myrtillocactus geotropizans*	Mexico	sandy loam natural sites	Illumina MiSeq	V4	Proteobacteria	54%	Fonseca-García et al. (2016)
						Actinobacteria	23%	
						Firmicutes	22%	
	*Opuntia robusta*					Proteobacteria	56%	
						Actinobacteria	22%	
						Bacteroidetes ^**c**^	9%	
						Firmicutes	8%	
	*Opuntia ficus-indica*	Tunisia	protected natural sites	Illumina MiSeq	V3-V4	Proteobacteria	49%	Karray et al. (2020)
						Actinobacteria	23%	
						Cyanobacteria	17%	
						Bacteroidetes	4%	
						Chloroflexi	2%	
						Firmicutes	2%	
						Saccharibacteria	2%	
	*Salicornia europea*	Poland	natural and anthropogenic saline sites	Illumina MiSeq	V3-V4	Proteobacteria	72%	Szymańska et al. (2018)
						Bacteroidetes	9%	
						Firmicutes	2%	
						Planctomycetes	2%	
Cycadales	*Cycas debaoensis *(normal roots)	China	botanical garden	Illumina HiSeq	V1-V9	Proteobacteria	76%	Pecundo et al. (2021)
						Actinobacteria	19%	
						Planctomycetes	5%	
	*C. debaoensis *(coralloid roots)					Cyanobacteria	91%	
						Proteobacteria	7%	
	*Cycas panzhihuaensis *(coralloid roots)	China	botanical garden and natural sites	Illumina MiSeq	V4-V5	Actinobacteria	53%	Zheng and Gong (2019)
						Proteobacteria	32%	
						Firmicutes	2%	
Malpighiales	*Populus alba *x* P. belorinensis *(non-transgenic)	China	saline and non-saline sites	Illumina MiSeq	V5-V7	Actinobacteria	56%	Wang et al. (2019)
						Proteobacteria	39%	
						Bacteroidetes	2%	
						Firmicutes	2%	
	*Populus deltoides*	USA	upland and bottomland sandy loam / clay loam / clay sites	Roche 454	V4	Proteobacteria	82%	Gottel et al. (2011)
						Acidobacteria	7%	
						Firmicutes	4%	
						Verrucomicrobia	3%	
						Actinobacteria	2%	
	*Populus deltoides *and *P. deltoides *x *P. trichocarpa*	USA	riparian habitat soils	Roche 454	V4	Proteobacteria	50%	Bonito et al. (2019)
						Actinobacteria	23%	
						Bacteroidetes	19%	
Fabales	*Melilotus albus*	Canada	oil sand reclamation site	Illumina MiSeq	V4	Proteobacteria	85%	Mitter et al. (2017)
						Actinobacteria	8%	
						Bacteroidetes	4%	
						Firmicutes	3%	
Poales	*Hordeum vulgare*					Proteobacteria	55%	
						Actinobacteria	24%	
						Tenericutes	13%	
						Bacteroidetes	6%	
	*Hordeum vulgare*	Germany	research field soil	Roche 454	V5-V7	Proteobacteria	61%	Bulgarelli et al. (2015)
						Bacteroidetes	20%	
						Actinobacteria	15%	
						Chloroflexi	3%	
	*Oryza sativa* and *O. glaberrima*	USA	rice field soils	Illumina MiSeq	V4	Proteobacteria	54%	Edwards et al. (2015)
						Chloroflexi	17%	
						Acidobacteria	5%	
						Bacteroidetes	4%	
						Fibrobacteres	4%	
						Spirochaetes	4%	
						Actinobacteria	3%	
						Firmicutes	3%	
						Verrucomicrobia	2%	
	*Panicum virgatum *and *P. hallii*	USA	natural research field site	Illumina MiSeq	V4	Proteobacteria	44%	Singer et al. (2019)
						Actinobacteria	35%	
						Bacteroidetes	7%	
						Firmicutes	5%	
						Chloroflexi	2%	
	9 C3 Poaceae sp.	USA	silty and sandy loam agricultural sites	Illumina MiSeq	V3-V4	Proteobacteria	52%	^**d**^Naylor et al. (2017)
						Bacteroidetes ^**c**^	26%	
						Actinobacteria	8%	
	9 C4 Poaceae sp.					Proteobacteria	43%	
						Bacteroidetes ^**c**^	28%	
						Actinobacteria	10%	
						Acidobacteria	3%	
	*Triticum turgidum *ssp.* durum*	Italy	sandy loam agricultural soil	Illumina MiSeq	V1-V3	Proteobacteria	76%	^**d**^Agnolucci et al. (2019)
						Actinobacteria	10%	
						Bacteroidetes	4%	
						Tenericutes	3%	
Solanales	*Capsicum annuum*	Mexico	agricultural fields	Illumina MiSeq	V3-V4	Proteobacteria	46%	Barraza et al. (2020)
						Firmicutes	19%	
						Bacteroidetes	13%	
						Actinobacteria	10%	
						Acidobacteria	4%	
						Verrucomicrobia	2%	
	*Solanum lycopersicum*					Proteobacteria	51%	
						Cyanobacteria	24%	
						Actinobacteria	6%	
						Bacteroidetes	6%	
						Firmicutes	6%	
	*Solanum lycopersicum*	South Korea	greenhouse plantations	Illumina MiSeq	V5-V7	Proteobacteria	61%	Lee et al. (2019)
						Actinobacteria	14%	
						Firmicutes	3%	
						Bacteroidetes	2%	
	*Solanum tuberosum*	USA	sandy loam agricultural site	Roche 454	V1-V2	Proteobacteria	54%	Manter et al. (2010)
						Bacteroidetes	30%	
						Actinobacteria	5%	
						Acidobacteria	2%	
Vitales	*Vitis vinifera *and *V. riparia *x *V. berlandieri*	Italy	clay-rich vineyard	Illumina MiSeq	V3-V4	Proteobacteria	60%	Marasco et al. (2018)
						Actinobacteria	20%	
						Tenericutes	11%	
						Bacteroidetes	4%	

^a^In case of multiple sampling sites, soil types, or sampling seasons, the means of relative abundance values were used. In case of multiple genotypes, ecotypes, varieties, or closely related species, the means of relative abundance values were used

^b^*Arabidopsis thaliana* is a non-mycorrhizal plant species

^c^In the original article assigned to Sphingobacteria, here named Bacteroidetes, based on the current List of Prokaryotic names with Standing in Nomenclature (LPSN; lpsn.dsmz.de)

^d^Means of relative abundance values were obtained from untreated or control sample groups

Not only the genotype, but also the phenological stage of the host plant may cause variability in the composition of root endophytic bacterial communities (Van Overbeek and Van Elsas 2008). Such an effect was clearly demonstrated in field-grown durum wheat, where bacterial taxa affiliated with Firmicutes showed fluctuating relative abundance in roots and other plant organs during the growing season (Minervini et al. 2015). Likewise, endophytic bacterial community compositions changed significantly across the growing stages in roots of sweet potato (Marques et al. 2015) and of non-mycorrhizal sugar beet (Shi et al. 2014).

Additional studies have revealed significant shifts in the composition and function of root bacterial microbiota as an effect of environmental variability. Soil type and geographic location contribute variability to potential soilborne colonizers causing significant differences in the quantitative and qualitative composition of root endophytic bacterial communities (Conn and Franco 2004; Lundberg et al. 2012; Schlaeppi et al. 2014; Edwards et al. 2015; Hameed et al. 2015). Other abiotic factors, such as stress (Naylor et al. 2017), flooding (Ferrando and Scavino 2015), suboptimal mineral nutrition (Hameed et al. 2015), seasons (Mocali et al. 2003), or agricultural management practices (Seghers et al. 2004) also have been identified as drivers of root endophytic bacterial community changes.

Regarding the taxonomic position of members of root bacterial microbiota, Liu et al. (2017b) reviewed previously published datasets, revealing that the main phyla were represented by Proteobacteria (ca. 50% in relative abundance), Actinobacteria (ca. 10%), Firmicutes (ca. 10%), and Bacteroidetes (ca. 10%). They also reported that Chloroflexi, Cyanobacteria, Planctomycetes, Verrucomicrobia, Nitrospirae, and Armatimonadetes were common in root tissues, while others, for example, Acidobacteria and Gemmatimonadetes, almost were excluded from the root endosphere. As previously mentioned, however, several studies have confirmed the active roles of host plants in the recruitment of selected bacteria from the nearby soil environment. For example, the genera *Enterobacter*, *Pseudomonas*, and *Stenotrophomonas* (Proteobacteria) represented the core bacterial endophytes in the roots of sweet potato and rice (Marques et al. 2015; Sessitsch et al. 2012). Accordingly, *Pseudomonas*-like OTUs dominated in the roots of *Populus deltoides* (34%) (Gottel et al. 2011), while Proteobacteria, Actinobacteria, and Bacteroidetes were the dominating phyla in root bacterial communities of *A. thaliana* (Bulgarelli et al. 2012; Lundberg et al. 2012; Bodenhausen et al. 2013), wheat, and tomato (Liu et al. 2017a; Lee et al. 2019). In addition to such taxa, Firmicutes occurred in the roots of *Aloe vera* and *Capsicum annuum* (Akinsanya et al. 2015; Barraza et al. 2020). Overall, the strong selection by the host plant results in recruitment of an endophytic bacterial community much simpler than that of the rhizosphere and of the nearby soil environment (Novello et al. 2017). 

## Diversity and functionality of AMF-associated bacterial communities

The services provided by AMF often are facilitated by the large and diverse beneficial bacterial communities living closely associated with spores, sporocarps, and extraradical mycelium, frequently embedded in spore wall layers and, in sporocarpic species, in the microniches formed by peridial hyphae (Walley and Germida 1996; Filippi et al. 1998; Iffis et al. 2014) (Table 2). The mycorrhizosphere microbiota show diverse functional activities, ranging from the role of “mycorrhiza helper” (MH) to that of “plant growth promoter” (PGP). MH bacteria (MHB) can promote spore germination, mycelial growth, and mycorrhiza establishment, while PGP bacteria (PGPB) have the ability to enhance plant growth, nutrition, health, and stress resistance (Barea et al. 2002; Frey-Klett et al. 2007). In addition, some components of such beneficial microbiota possess both MH and PGP traits (Xavier and Germida 2003; Battini et al. 2017). Overall, MHB and PGPB show activities promoting and complementing those of AMF (Turrini et al. 2018; Giovannini et al. 2020). The metabolic traits underlying MH functions include growth factor production and detoxification of antagonistic substances, while PGP properties can range from N_2_ fixation, nutrient mobilization, and nutrient uptake facilitation to plant hormone, antibiotic, and siderophore production, or systemic resistance induction (Frey-Klett et al. 2007; Hayat et al. 2010).

**Table 2:** Bacterial communities closely associated with spores, sporocarps, extraradical mycelium and intraradical propagules of AMF species and isolates from different geographic locations

AMF species/isolate	Geographic location	Methodology	AMF-associated bacterial communities^a^	References
*Glomus versiforme* ^**b**^ spores	Department of Plant Pathology, Kanas State University, Manhattan, USA	Isolation in pure culture + morphological identification	*Corynebacterium* (Actinobacteria), *Pseudomonas* (Proteobacteria)	Mayo et al. (1986)
*G. clarum *NT4 spores	Saskatchewan, Canada	Isolation in pure culture + FAME profiles	*Arthrobacter ilicis *(Actinobacteria)*, Bacillus alvei, Bacillus brevis, Bacillus chitinosporus, Bacillus circulans, Bacillus firmus, Bacillus laterosporus, Bacillus longisporus, Bacillus megaterium, Bacillus pabuli *(Firmicutes)	Xavier and Germida (2003)
*Glomus geosporum* BEG 18 spores	Calcareous grassland at Nenzlingen, Switzerland	PCR-DGGE analysis	*Flexibacter (Bacteroidetes)*, *Cyanobacteria*, *Fibrobacteres Burkholderia, Cellvibrio*, *Chondromyces, Desulfovibrio, Lysobacter, Rheinheimera *(Proteobacteria)	Roesti et al. ( 2005)
*Glomus constrictum* BEG 19 spores	Id.^**c**^	Id.	*Flexibacter (Bacteroidetes)*, *Cyanobacteria*, *Fibrobacteres*, *Burkholderia*, *Cellvibrio*, *Chondromyces, Desulfovibrio*, *Lysobacter, Pseudomonas, Rheinheimera (Proteobacteria)*	Id.
*Gigaspora margarita* spores	Commercial inoculum at Central Glass Co., Tokyo, Japan	Isolation in pure culture	*Paenibacillus polymyxa *(Firmicutes), *Janthinobacterium lividum *(Proteobacteria)	Cruz et al. (2008)
*Glomus mosseae* spores	BIODEPTH site, Umeå, Sweden	FAME profiles	Cellulomonadaceae, Microbacteriaceae, Micrococcaceae (Actinobacteria), Bacillaceae (Firmicutes), Burkholderiaceae, Comamonadaceae, Pseudomonadaceae, Rhizobiaceae, Xanthomonadaceae (Proteobacteria)	Bharadwaj et al. (2008b)
*Glomus intraradices* spores	Id.	Id.	Cellulomonadaceae, Corynebacteriaceae, Micrococcaceae, Microbacteriaceae (Actinobacteria), Bacillaceae (Firmicutes), Alcaligenaceae, Oxalobacteraceae, Rhizobiaceae (Proteobacteria)	Id.
*Gigaspora margarita *MAFF 520054 spores	Ministry of Agriculture, Forestry and Fisheries Gene bank, Tsukuba, Japan	PCR-DGGE analysis	*Amycolatopsis, Pseudonocardia,* *Streptomyces *(Actinobacteria),* Flexibacter* (Bacteroidetes), *Azovibrio, Azospirillum, Polyangium cellulosum, Ramlibacter,* *Rhizobium, Rubrivivax, Sphingomonas *(Proteobacteria)	Long et al. (2008)
*Glomus irregulare* spores	Mirabel–Lachute, Québec, Canada	Isolation in pure culture + 16S rRNA gene sequencing	*Kocuria rhizophila, Microbacterium ginsengisoli* (Actinobacteria), *Bacillus cereus, Bacillus megaterium, Bacillus simplex* (Firmicutes), *Sphingomonas* sp., *Variovorax paradoxus* (Proteobacteria)	Lecomte et al. (2011)
*Gigaspora margarita *spores	Silviculture Laboratory, Faculty of Forestry, Institut Pertanian Bogor, Indonesia	Isolation in pure culture + 16S rRNA gene sequencing	*Bacillus* sp., *Bacillus flexus,* *Bacillus megaterium*, *Bacillus subtilis* (Firmicutes)	Budi et al. (2012)
*Gigaspora margarita* spores	Central Glass Co. Ltd, Tokyo, Japan	Isolation in pure culture + 16S rRNA gene sequencing	*Bacillus* sp., *Bacillus thuringiensis, Paenibacillus* *rhizospherae* (Firmicutes)	Cruz & Ishii (2011)
Intraradical AMF structures (*Diversispora eburnea*, *Archaeospora schenckii*, *Glomus *sp.,* Claroideoglomus *sp.,* Glomus irregulare*)	St-Lawrence River, Montreal, Quebec, Canada	16S rRNA gene cloning and sequencing	*Propionibacterium* (Actinobacteria), *Bacillus, Paenibacillus, Streptococcus, Lactobacillus* (Firmicutes), *Acinetobacter*, *Afipia*, *Agrobacterium*, *Azospirillum*, *Bosea*, *Bradyrhizobium*, *Brevundimonas, Legionella*,* Leptothrix*, *Lysobacter*, *Massilia*, *Methylobacterium*, *Pseudoacidovorax*, *Pseudomonas*, *Pseudoxanthomonas,* *Sphingomonas*, *Stenotrophomonas *(Proteobacteria)	Iffis et al. (2014)
*Funneliformis coronatum *IMA3 spores	Microbiology Labs, Department of Agricultural, Food and Environment, University of Pisa, Italy	PCR-DGGE analysis	*Arthrobacter *(Actinobacteria),* Agrobacterium, Sinorhizobium *(Proteobacteria)*,* Mollicutes related endobacteria (Mre)	Agnolucci et al. (2015)
*Funneliformis mosseae *AZ225C spores	Id.	Id.	Acidobacteria, *Arthrobacter, Streptomyces *(Actinobacteria)*, *Bacteroidetes,* Paenibacillus *(Firmicutes), * Agrobacterium, Duganella, Herbaspirillum, Ideonella, Sinorhizobium, Rhizobium *(Proteobacteria)	Id.
*Funneliformis mosseae *IN101C spores	Id.	Id.	*Propionibacterium (Actinobacteria)*, *Methylibium (Proteobacteria)*, Mre	Id.
*Funneliformis mosseae *IMA1spores	Id.	Id.	*Uncultured Bacteroidetes, Uncultured Deltaproteobacteria, *Mre,* Amycolatopsis*	Id.
*Rhizophagus intraradices* IMA5 spores	Id.	Id.	*Streptomyces* (Actinobacteria), Uncultured Deltaproteobacteria*, Pseudomonas, Sinorhizobium* (Proteobacteria), Mre	Id.
*Rhizophagus intraradices* IMA6 spores	Id.	Id.	*Arthrobacter, Streptomyces *(Actinobacteria), *Bacillus *(Firmicutes),* Herbaspirillum, Massilia, Pseudomonas, Rhizobium *(Proteobacteria)	Id.
*Rhizophagus intraradices* IMA6 spores	Microbiology Labs, Department of Agricultural, Food and Environment, University of Pisa, Italy	Isolation in pure culture + 16S rRNA gene sequencing	*Arthrobacter phenanthrenivorans, Nocardioides albus, Streptomyces* (Actinobacteria), *Bacillus pumilus, Fictibacillus, Lysinibacillus fusiformis* (Firmicutes)*, Sinorhizobium meliloti *(Proteobacteria)	Battini et al. (2016)
*Gigaspora margarita* J5 spores	BGIV collection School of Exact and Natural Sciences, University of Buenos Aires, Argentina	Isolation in pure culture + 16S rRNA gene sequencing	*Bacillus megaterium* (Firmicutes), *Azospiriullum, Pandoraea, Pseudomonas Rhizobium etli, Stenotrophomonas maltophila *(Proteobacteria)	Bidondo et al. (2016)
*Funneliformis mosseae* G1 sporocarps	Id.	Id.	*Bacillus, Paenibacillus favisporus, Paenibacillus *spp. (Firmicutes)	Id.
*Rhizophagus-Glomus* spp. intraradical structures	Id.	Id.	*Bacillus, Cohnella, Paenibacillus rhizosphaerae *(Firmicutes), *Pseudomonas* (Proteobacteria)	Id.
AMF spores from the field	St-Lawrence River, Montreal, Canada	16S rRNA gene 454 sequencing	Gammaproteobacteria (49%) Betaproteobacteria (23%) Alphaproteobacteria (6%)	Iffis et al. (2016)
			Actinobacteria (11%)	
*Rhizophagus intraradices* spores	Saemangeum reclamation land, South Korea	Isolation in pure culture + 16S rRNA gene sequencing	*Massilia *sp. (Proteobacteria)	Krishnamoorthy et al. (2016)
*Funneliformis caledonium *spores	Saemangeum, South Korea	Isolation in pure culture + 16S rRNA gene sequencing	*Bacillus aryabhattai* (Firmicutes)	Selvakumar et al. (2016)
*Racocetra alborosea *spores	Id.	Id.	*Bacillus anthracis, Bacillus aryabhattai, Paenibacillus xylanexedens* (Firmicutes)	Id.
*Funneliformis mosseae *spores	Id.	Id.	*Bacillus anthracis, Bacillus aryabhattai* (Firmicutes)	Id.
*Gigaspora margarita *spores	Ministry of Agriculture, Forestry, and Fisheries Gene bank, Tsukuba, Japan	Isolation in pure culture + 16S rRNA gene sequencing	*Amycolatopsis, Arthrobacter, Curtobacterium, Gordonia, Leifsonia, Mycobacterium, Nocardia, Streptomyces* (Actinobacteria), *Bacillus, Brevibacillus Paenibacillus* (Firmicutes), *Achromobacter, Aquitalea, Bosea, Burkholderia, Cupriavidus, Ensifer, Lysobacter, Mitsuaria, Proteus, Pseudomonas, Ralstonia, Rhizobium *(Proteobacteria)	Long et al. (2017)
*Funneliformis mosseae* CMU-RYA08 spores	Rayong Province, Thailand	Isolation in pure culture + 16S rRNA gene sequencing	*Pseudonocardia nantongensis,* *Streptomyces pilosus, Streptomyces spinoverrucosus, Streptomyces thermocarboxydus *(Actinobacteria)	Lasudee et al. (2018)
*Rhizoglomus irregulare* BEG72 commercial inoculum	Atens, Agrotecnologias Naturales S.L., La Riera de Gaia, Tarragona, Spain	Isolation in pure culture + 16S rRNA gene sequencing	*Microbacterium trichotecenolyticum,* *Streptomyces* (Actinobacteria) *Bacillus litoralis, Bacillus megaterium* (Firmicutes), *Enterobacter, Rhizobium radiobacter* (Proteobacteria)	Agnolucci et al. (2019)
Id.	Id.	16S rRNA gene Illumina sequencing	Proteobacteria (37%)	Id.
			Bacteroidetes (29%)	
			Actinobacteria (8%)	
			Planctomycetes (6%)	
			Verrucomicrobia (4%)	
			Firmicutes (3%)	
			Deinococcus-Thermus (3%)	
			Patescibacteria (3%)	
			Fibrobacteres (2%)	
*Rhizoglomus irregulare* QS69 extraradical hyphae	INOQ GmbH, Schnega, Germany	Isolation in pure culture + 16S rRNA gene sequencing	*Ochrobactrum anthropi,* *Pseudomonas fluorescens, Pseudomonas putida, Pseudomonas fuscovaginae, Pseudomonas koreensis *(Proteobacteria)	Sharma et al. (2020)
*Glomus versiforme* extraradical hyphae	INVAM collection	16S rRNA gene Illumina sequencing	Proteobacteria (50%)	Emmett et al. (2021)
			Actinobacteria (10%)	
			Chloroflexi (9%)	
			Acidobacteria (7%)	
			Bacteroidetes (6%)	
			Fibrobacteres (4%)	

^a^Taxa are listed in alphabetical order when quantitative data are not available

^b^The original nomenclature of AMF has been retained here for proper cross-reference to previous works

^c^Id.: same as above (Idem)

From a taxonomic viewpoint, the composition of AMF-associated bacterial microbiota strongly depends on AMF identity. Indeed, PCR-DGGE analysis of 16S rDNA showed that the bacterial communities associated with AMF spores were more influenced by fungal than host plant species. Overall, PCR-DGGE allowed the detection of bacterial sequences affiliated with the genera *Cellvibrio*, *Chondromyces*, *Lysobacter*, *Pseudomonas* (Proteobacteria), and *Flexibacter* (Bacteroidetes)*.* Such bacteria, in particular the genus *Flexibacter*, well known for their ability to degrade biopolymers, were suspected of feeding on the spore wall, which consists mainly of chitin (Roesti et al. 2005). The same molecular approach revealed differences in the composition of spore-associated bacterial communities of two AMF, *Gigaspora margarita* and *Gigaspora rosea*, and showed that most of the bacterial sequences from *G. margarita* were affiliated with Proteobacteria (*Azospirillum*, *Azovibrio*, *Polyangium*, *Ramlibacter*, *Rubrivivax*, *Sphingomonas*, *Rhizobium*) and Actinobacteria (*Streptomyces*, *Amycolatopsis*, and *Pseudonocardia*) (Long et al. 2008). Interestingly, by PCR-DGGE and band sequencing, Agnolucci et al. (2015) revealed that the spores of six different AMF harbored unique bacterial communities, which were not correlated with the taxonomic positions of the fungi. The sequences were affiliated with Actinomycetales, Bacillales, Burkholderiales, Pseudomonadales, and Rhizobiales, all orders encompassing taxa known as PGP bacteria. These three mentioned works reached consistent conclusions, suggesting that the differences in the composition of spore walls or spore exudates may affect the recruitment of spore-associated bacterial communities. In contrast, bacterial communities closely associated with AMF spores were reported to be shaped not only by fungal identity, but also by the identity of the host plant (Iffis et al. 2016). Gammaproteobacteria were more abundant in spores collected from *Solidago canadensis* soil samples than from *Populus balsamifera* and *Lycopus europaeus*, whereas spores belonging to the genus *Glomus* were correlated with Betaproteobacteria, Actinobacteria, Bacilli, and Sphingobacteria. In this case, the authors suggested that the strategy of differential bacteria recruitment by diverse AMF species and isolates also might reflect variations in the composition of spores/hyphae exudates, attracting specific microbial communities (Iffis et al. 2016). The underlying mechanisms of such differential recruitment among different AMF remain to be thoroughly investigated.

A novel study reported a surprisingly high diversity of bacteria associated with AMF vesicles and intraradical spores extracted from microdissected roots of *Solidago rugosa*. The dominant sequences belonged to the genera *Sphingomonas*, *Pseudomonas*, *Massilia*, and *Methylobacterium* (Proteobacteria) while *Bradyrhizobium*, *Bosea* (Proteobacteria), *Bacillus*, and *Paenibacillus* (Firmicutes) were found at lower frequencies (Iffis et al. 2014).

Only a limited number of studies have investigated the bacterial communities closely associated with the surface of AMF hyphae. One of the first works, by using bromodeoxyuridine immunocapture and confocal microscopy, determined the specific attachment of a strain of *Bacillus cereus* to AMF hyphae (Artursson and Jansson 2003). Toljander et al. (2006) reported that five different strains of *gfp*-tagged soil bacteria, inoculated into in vitro cultures of two *Glomus* isolates growing in a controlled artificial system – T-DNA-transformed roots – exhibited different levels of hyphal attachment. In particular, *Paenibacillus brasiliensis*, *Bacillus cereus*, *Paenibacillus peoriae* (Firmicutes), and *Pseudomonas fluorescens* (Proteobacteria) attached to AMF hyphae, while *Arthrobacter chlorophenolicus* (Actinobacteria) did not. Such differences can be ascribed to AMF hyphal exudates that have been reported to affect the composition of bacterial communities (Toljander et al. 2007). Similar in vitro experimental systems showed that *Streptomyces* and members of the Oxalobacteraceae family, i.e., *Duganella*, *Janthinobacterium*, and *Massilia*, were specifically attached to the surface of AMF hyphae (Scheublin et al. 2010). Interestingly, 26 *Burkholderia* spp. strains and one *Rhizobium miluonense* strain were able to strongly attach to *Rhizophagus irregularis* hyphae and to solubilize phosphate (Taktek et al. 2015). Consistent data were obtained by a recent work reporting the isolation of 128 bacterial strains from the hyphae of *Rhizoglomus irregulare* (syn. *Rhizophagus irregularis*), of which 12 showed phosphate-solubilizing activity (Sharma et al. 2020). A distinct bacterial community closely associated with extraradical hyphae of *Glomus versiforme*, and conserved across divergent soils, was mainly represented by Proteobacteria (50% relative abundance), Actinobacteria (10%), Chloroflexi (9%), Acidobacteria (7%), Bacteroidetes (6%), and Fibrobacteres (4%) (Emmett et al. 2021). An in vivo study reported, for the first time, that AMF hyphae may act as “transport agents” or “highways” for bacteria. Indeed, a *gfp*-tagged nitrogen-fixing rhizobial strain, *Bradyrhizobium diazoefficiens*, was able to tightly adhere to *Glomus formosanum* hyphae, facilitating bacterial translocation to their legume host plant and the formation of N-fixing nodules in the root system (de Novais et al. 2020). Recently, Illumina MiSeq metagenome sequencing allowed the identification of 276 bacterial genera, belonging to 165 families, 107 orders, and 23 phyla, mostly represented by Proteobacteria, Bacteroidetes, and Actinobacteria, associated with *Rhizoglomus irregulare* commercial inoculum. Such richness and diversity are remarkable, given that no bacteria were deliberately added to the AM symbiont. It is interesting to note that the predominant bacterial taxa correspond to those recurrently found in the root endosphere of the majority of plant species investigated so far (Table 1).

Culture-dependent analyses not only confirmed the high diversity of bacterial communities living in association with AMF, but also showed the PGP activity of strains belonging to Actinomycetales, Bacillales, Enterobacteriales, and Rhizobiales, as IAA and siderophore producers (Agnolucci et al. 2019a). Such activities are important for plant development because the phytohormone IAA is able to modulate the growth and functioning of the root system (Duca et al. 2014), while siderophores, high-affinity iron-chelating compounds, may facilitate plant iron acquisition and control soilborne diseases by means of iron competition (Mimmo et al. 2014). Other culture-based studies reported a high abundance of the mentioned phyla, recording genera such as *Micrococcus*, *Acidovorax*, *Cellulomonas*, *Janthinobacterium*, *Alcaligenes*, and *Flavobacterium* (Xavier and Germida 2003; Bharadwaj et al. 2008b; Cruz et al. 2008). Some bacteria with PGP potentials, isolated from the spores of *Rhizophagus intraradices*, affiliated with the genera *Sinorhizobium*/*Ensifer*, *Streptomyces*, *Bacillus*, *Arthrobacter*, and *Fictibacillus*, also have shown MH properties (Battini et al. 2016). Indeed, seven of such bacterial isolates significantly increased hyphal length density, while two of them, *Streptomyces* sp. W77 and *Streptomyces* sp. W94, additionally were able to promote specific P uptake and translocation in maize plants (Battini et al. 2017).

Overall, the available data show that diverse bacterial taxa are differentially able to attach to AMF structures, i.e., spores, sporocarps, and hyphae, of which exudates might differ in quantity and/or quality, thus promoting or inhibiting the growth and attachment of particular bacterial communities. It is important to note that such specific and close physical relationships may be indicative of complex interactions among AMF, bacteria, and host plants, suggesting that AMF might act as carriers of the endophytic microbiota that establish in roots.

## Concluding observations and future prospects

The role of AMF as drivers of the endophytic bacterial communities colonizing plant roots can be revealed by investigating possible overlaps in the taxonomic composition of root endophytic microbiota and AMF-associated bacterial communities. Only a few such comparisons currently are available, but major shifts have been revealed in the composition of the root endophytic bacterial communities of durum wheat after inoculation with the AM symbiont *Funneliformis mosseae*. The use of two culture-independent approaches, PCR-DGGE analysis of 16S rDNA and high-throughput sequencing of the same gene through Illumina MiSeq, has revealed that AMF inoculation increased the abundance of some genera and species of Actinobacteria and Bacteroidetes in two durum wheat cultivars, Odisseo and Saragolla (Agnolucci et al. 2019b). In particular, *Funneliformis mosseae* increased the abundance of Actinobacteria, such as *Rhodococcus* species, in the cv. Saragolla, and that of *Streptomyces* and *Microbacterium* spp. in both cultivars. This is an interesting finding, as Actinobacteria, considered promising PGP bacteria (Seipke et al. 2012), have been reported to be closely associated with the spores of six different AMF isolates (Agnolucci et al. 2015). Moreover, in the two durum wheat cultivars, AMF inoculation increased the abundance of the Proteobacteria *Pantoea*, a PGP genus, including species and strains able to produce siderophores, enhance Zn availability in soil, and induce plant resistance to drought stress (Moreira et al. 2016; Kamran et al. 2017; Chen et al. 2017).

The possible mechanisms operating in the recruitment of different bacterial endophytic communities in roots may be dual. As the physiological status of plants is altered when they are mycorrhizal (e.g., photosynthesis, nutrition and growth, phenology, health) (Smith and Read 2008), certain groups of endophytic bacteria might find specific conditions best suited for their growth and development and thus might be favored in colonization of such a privileged ecological niche. On the other hand, they may gain facilitated access to root tissues during AMF establishment through their close association with AMF hyphae (Toljander et al. 2006; de Novais et al. 2020). These two mechanisms may act simultaneously, and it will be difficult to discriminate between them. Notwithstanding, systematic studies on the differential occurrence of root bacterial endophytes in mycorrhizal and non-mycorrhizal plants still are needed.

In the years to come, a series of key questions remain to be answered. The first question concerns whether members of AMF-associated bacterial communities can actually establish in the root system, becoming endophytic, and in what proportion. If so, would they be able to show the specific PGP properties revealed when isolated and grown in vitro? The results obtained will raise the question as to whether AMF identity and diversity can affect the structure and composition of root endophytic bacterial communities. Moreover, as AMF commercial inocula carry their own associated bacterial microbiota, could they represent suitable tools to introduce beneficial root endophytes in sustainable agriculture? Such questions can only be answered by targeted research on the diversity and functionality of root endophytic communities as affected by the presence of AMF and AMF-associated bacterial communities.
